# Morphological and cytological observations of corolla green spots reveal the presence of functional chloroplasts in Japanese gentian

**DOI:** 10.1371/journal.pone.0237173

**Published:** 2020-08-26

**Authors:** Shigekazu Takahashi, Suguru Ozawa, Kintake Sonoike, Katsutomo Sasaki, Masahiro Nishihara

**Affiliations:** 1 Iwate Biotechnology Research Center, Kitakami, Iwate, Japan; 2 Iwate Agricultural Research Center, Kitakami, Iwate, Japan; 3 Faculty of Education and Integrated Arts and Sciences, Waseda University, Shinjuku-ku, Tokyo, Japan; 4 Institute of Vegetable and Floriculture Science, National Agriculture and Food Research Organization (NARO), Tsukuba, Ibaraki, Japan; University of Tsukuba, JAPAN

## Abstract

Gentian is an important ornamental flower in Japan. The corolla of the majority of cultivated Japanese gentians have green spots, which are rarely encountered in flowers of other angiosperms. Little information is available on the functional traits of the green spots. In this study, we characterized the green spots in the Japanese gentian corolla using a number of microscopic techniques. Opto-digital microscopy revealed that a single visible green spot is composed of approximately 100 epidermal cells. The epidermal cells of a green spot formed a dome-like structure and the cell lumen contained many green structures that were granular and approximately 5 μm in diameter. The green structures emitted red autofluorescence when irradiated with 488 nm excitation light. Transmission electron microscopy revealed that the green structures contained typical thylakoids and grana, thus indicating they are chloroplasts. No grana were observed and the thylakoids had collapsed in the plastids of epidermal cells surrounding green spots. To estimate the rate of photosynthetic electron transfer of the green spots, we measured chlorophyll fluorescence using the MICROSCOPY version of an Imaging-PAM (pulse-amplitude-modulated) fluorometer. Under actinic light of 449 μmol m^−2^ s^−1^, substantial electron flow through photosystem II was observed. Observation of green spot formation during corolla development revealed that immature green spots formed at an early bud stage and developed to maturity associated with chloroplast degradation in the surrounding epidermal cells. These results confirmed that the Japanese gentian corolla contains functional chloroplasts in restricted areas of epidermal cells and indicated that a sophisticated program for differential regulation of chloroplast formation and degradation is operative in the epidermis.

## Introduction

Angiosperms have evolved a variety of flower traits, such as diverse colors, patterns, shapes, and scents, to attract pollinators for efficient sexual reproduction [1, 2]. Among angiosperms, *Gentiana* is a genus of flowering plants belonging to the gentian family (Gentianaceae). More than 400 species are classified in this cosmopolitan genus and are specially distributed in alpine areas [3]. In Japan, gentians are important ornamental plants grown as a cut flower or a flowering potted plant. Furthermore, the roots and rhizomes of gentians are used in traditional medicine for skin diseases caused by wind-heat or damp-heat [4]. To date, more than 300 gentian cultivars derived from *G*. *scabra*, *G*. *triflora*, and their hybrids have been bred and selected for ornamental purposes [5]. Generally, Japanese cultivated gentians have green spots on the corolla adaxial surface, irrespective of flower color (S1 Fig). Previous molecular biological studies have investigated diverse characteristics of Japanese gentians, which include not only flower color (detailed in latter part) but also flower shape [6, 7], flowering time [8], overwintering [9], and floral odor [10].

The vivid blue color of the corolla of Japanese gentians is conferred by gentiodelphin, a polyacylated anthocyanin [11]. The structure of other delphinidin derivatives has also been characterized [12]. Several Japanese gentians produce pink or white flowers (S1 Fig). Previous research revealed that disruption of the gene encoding flavonoid 3′,5′-hydroxylase (*GtF3′5′H)*, a crucial enzyme for delphinidin biosynthesis, results in development of a pink corolla in the mutant [13, 14]. In a similar manner, disruption of the genes that encode anthocyanidin synthase (*GtANS*) and a R2R3-MYB transcription factor (*GtMYB3*) regulating expression of genes associated with flavonoid biosynthesis leads to a white-corolla phenotype [15, 16]. Using this information, molecular DNA markers to distinguish individuals with pink or white corollas from genotypes with blue-pigmented corollas have been developed [17, 18]. Recently, novel technologies, such as radiation with heavy ion beams and genome editing using the clustered regularly interspaced short palindromic repeats (CRISPR)/CRISPR-associated protein 9 (CRISPR/Cas9) system, have been applied to induce novel flower colors in gentian [19, 20].

In contrast to the studies of flower color, no scientific research has been conducted previously on the green spots of the Japanese gentian corolla to the best of our knowledge. On the other hand, in other plant families, several studies on the formation of petal spots have been performed and are well summarized in a review by Davies et al. [1]. For instance, in *Iris japonica*, the accumulation of carotenoids, anthocyanins or both in epidermal cells form spots. In Asiatic hybrid lilies (*Lilium* spp.; Liliaceae), splatter-type spots are formed via anthocyanin accumulation regulated by LhMYB12-Lat expression [21]. In *Clarkia gracilis* (Onagraceae), the anthocyanin biosynthetic pathway is regulated to form sector-type spots [22]. *Swertia bimaculata* (Gentianaceae) possesses nectaries, with a flat spot-like shape and yellow-green color, that are located in the middle of the corolla lobe; it is reported that the nectaries function as pollinator manipulators [23].

The presence of green spots on the corolla is an important factor that determines the commercial value of Japanese gentians. Green spots are usually an undesirable trait and breeders select lines with few green spots on the corolla. The size and number of the green spots vary among cultivars, and are barely noticeable in some cultivars. Japanese gentians generally do not bloom in the year of planting, therefore molecular markers are extremely useful to expedite gentian breeding [5, 24]. Accurate prediction of the presence or absence of green spots before flowering using DNA markers will greatly improve breeding efficiency. To this purpose, we aim to develop DNA markers for green spots. However, limited information on corolla green spots in gentian is available and detailed observation has not been conducted. In this study, we focused on green spots of the gentian corolla and aimed to characterize their properties using a variety of microscopic techniques. We determined that the cells in green spots retain functional chloroplasts, unlike the surrounding epidermal cells. This is the first report describing the traits of green spots on the corolla of Japanese gentians.

## Materials and methods

### Plant materials

The Japanese gentian breeding line ‘Bzc-1’ [19] was used in this study. The plants were grown in soil in pots under natural daylight in a greenhouse at the Iwate Agricultural Research Center (S2 Fig).

### Opto-digital microscopy

The epidermis and cross-sections of the ‘Bzc-1’ corolla with green spots were observed with an opto-digital microscope (DSX500, Olympus Co., Tokyo, Japan). The cross-sections were cut by hand to 300 μm thickness with a razor blade. A digital microscopy image of the corolla used for SEM analysis was taken by Leica MZ16 FA (Leica microsystems, Wetzlar, Germany) with DIGITAL SIGHT DS-5Mc (Nikon, Kawasaki, Japan).

### Scanning electron microscopy

**Fig 1:**
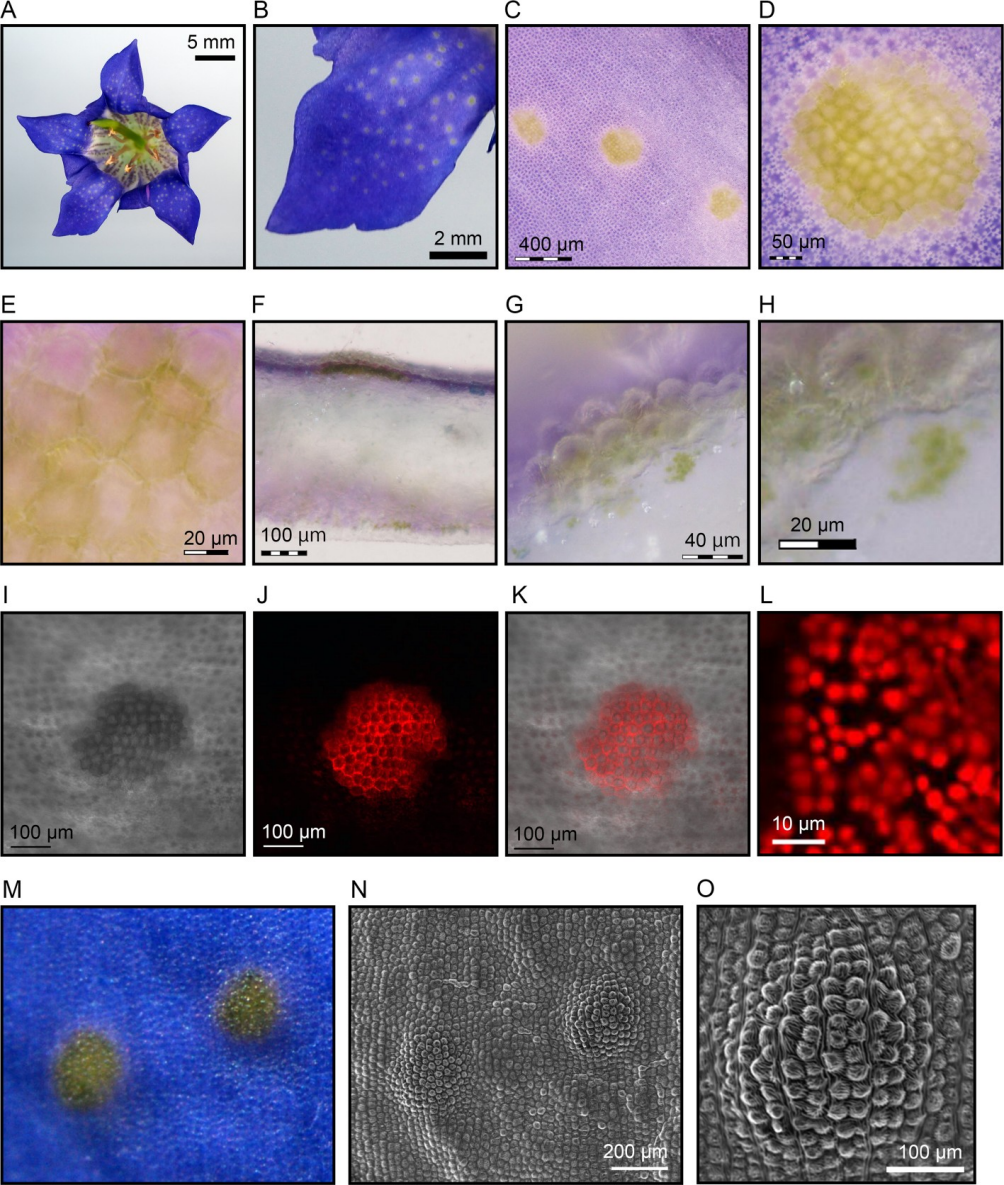
Morphology and anatomy of the corolla of Japanese gentian ‘Bzc-1’. (A) Flower morphology, (B) adaxial surface of the corolla, (C) high-magnification image of the adaxial surface of the corolla, (D) high-magnification image of a green spot, (E) green epidermal cells (GECs), (F) cross-section of a corolla, (G) cross-section of a green spot, (H) cross-section of GECs. (I–L) Confocal laser micrographs of green spots. The images in (I), (J), and (K) correspond to a Nomarski differential interference contrast image, autofluorescence, and the merged image, respectively. Autofluorescence of GECs is shown in (L). (M) A digital microscopy image of the corolla used for SEM analysis, (N) SEM image of the adaxial surface of the corolla, (O) SEM image of the adaxial surface of a green spot.

Fresh corollas (without fixation) with green spots sampled from ‘Bzc-1’ were subjected to scanning electron microscopy (SEM) analysis. The SEM images shown in Fig 1 were acquired using a JCM-6000 NeoScope (JEOL Ltd, Tokyo, Japan) at an acceleration voltage of 15 kV. The SEM images shown in Fig 4 were captured using a Miniscope TM4000 (Hitachi High-Tech Co., Tokyo, Japan) at an acceleration voltage of 15 kV.

### Confocal laser scanning microscopy

Fresh corollas sampled from ‘Bzc-1’ were observed with a FLUOVIEW FV1000 confocal laser scanning microscope (Olympus Co., Tokyo, Japan). Fluorescence images were acquired using a 650–750 nm long-pass filter after excitation with an Ar laser (λ_ex_ = 488 nm).

### Transmission electron microscopy

Fresh corollas sampled from ‘Bzc-1’ were fixed with 2% paraformaldehyde and 2% glutaraldehyde in 0.05 M cacodylate buffer (pH 7.4) at 4°C overnight. After fixation, the samples were washed three times with 0.05 M cacodylate buffer for 30 min each, and were postfixed with 2% osmium tetroxide in 0.05 M cacodylate buffer at 4°C for 3 h. Subsequently, the samples were dehydrated in a graded ethanol series (50%, 70%, 90%, and 100%). The dehydrated samples were infiltrated with propylene oxide (PO) twice for 30 min each and were placed into a 70:30 mixture of PO and resin (Quetol-651, Nisshin EM Co., Tokyo, Japan) for 1 h. Subsequently, the tube cap was opened and PO was volatilized overnight. The samples were transferred to fresh 100% resin and were polymerized at 60°C for 48 h. The polymerized resin blocks were ultra-thin-sectioned (80 nm) with a diamond knife using an Ultracut UCT ultramicrotome (Leica, Vienna, Austria). The sections were mounted on copper grids, stained with 2% uranyl acetate at room temperature for 15 min, washed with distilled water, then secondary-stained with Lead stain solution (Sigma-Aldrich Co., Tokyo, Japan) at room temperature for 3 min. The grids were observed using a transmission electron microscope (JEM-1400Plus, JEOL Ltd, Tokyo, Japan) at an acceleration voltage of 100 kV. Digital images were captured with a CCD camera.

### Imaging pulse-amplitude-modulated (PAM) chlorophyll fluorescence

Chlorophyll fluorescence imaging was performed using the MICROSCOPY version of Imaging-PAM (Heinz Walz, Effeltrich, Germany) fluorometer with a microscope (Axioscope with 10× objective lens, Carl Zeiss, Germany) and ImagingWinGigE software (Heinz Walz). A blue LED lamp (IMAG-L470M, Heinz Walz) was used for measurements or as an actinic light source. A small piece (about 1 cm^2^) of ‘Bzc-1’ corolla with green spots was placed on a microscope slide and dark-acclimated for 5 min at room temperature. Subsequently, images of Φ_II_, a parameter that represents photosynthetic yield (effective quantum yield of photosynthetic electron transfer), were obtained under actinic light at 449 μmol m^–2^ s^–1^.

## Results

### Structure of green spots on corolla

The breeding line ‘Bzc-1’, a typical Japanese gentian (producing flowers with a blue corolla and green spots), was selected as the study material (Fig 1A and 1B). ‘Bzc-1’ bears dozens of green spots per corolla. The green spots are present in the corolla lobe but not in the paracorolla. Surface images of a green spot acquired with an opto-digital microscope are shown in Fig 1C–1E. A green spot was composed of approximately 100 epidermal cells (Fig 1D). Furthermore, the epidermal cells forming a green spot contained many green objects (Fig 1E). To clarify the cell types that constituted the green spots, we prepared cross-sections of the ‘Bzc-1’ corolla and observed that the green spots were composed solely of epidermal cells (Fig 1F). The green objects had a granular structure approximately 5 μm in diameter (Fig 1G and 1H). Hereafter, the epidermal cells forming a green spot are referred to as green epidermal cells (GECs).

Next, the corolla of ‘Bzc-1’ was observed using a confocal laser microscope (Fig 1I–1L). Under 488 nm blue excitation light, the green spot emitted red autofluorescence (Fig 1J). In contrast, epidermal cells other than GECs did not autofluoresce (Fig 1J). Hereafter, epidermal cells other than GECs are referred to as blue epidermal cells (BECs) designated after the vivid blue pigmentation. Observation of the GECs at higher magnification revealed that the granular structures emitted red fluorescence (Fig 1L).

To reveal the surface structure of GECs and BECs, we next performed SEM analysis. Recently, Bailes and Glover classified the protruding parts of epidermal cell morphology of *Vicia faba* L. by perimeter shape, projection, and cell surface micromorphology [25]. According to their classification method, perimeter shape, projection, and cell surface micromorphology of GECs and BECs were categorized into papillose, conical and striate, respectively (Fig 1M–1O). In addition, a green spot formed a dome-like structure. Other than the formation of a dome, the surface structure of GECs and BECs did not differ. The physiological function of the dome is uncharacterized to date and may be a crucial feature to elucidate the role of green spots.

### GECs contain chloroplasts

**Fig 2:**
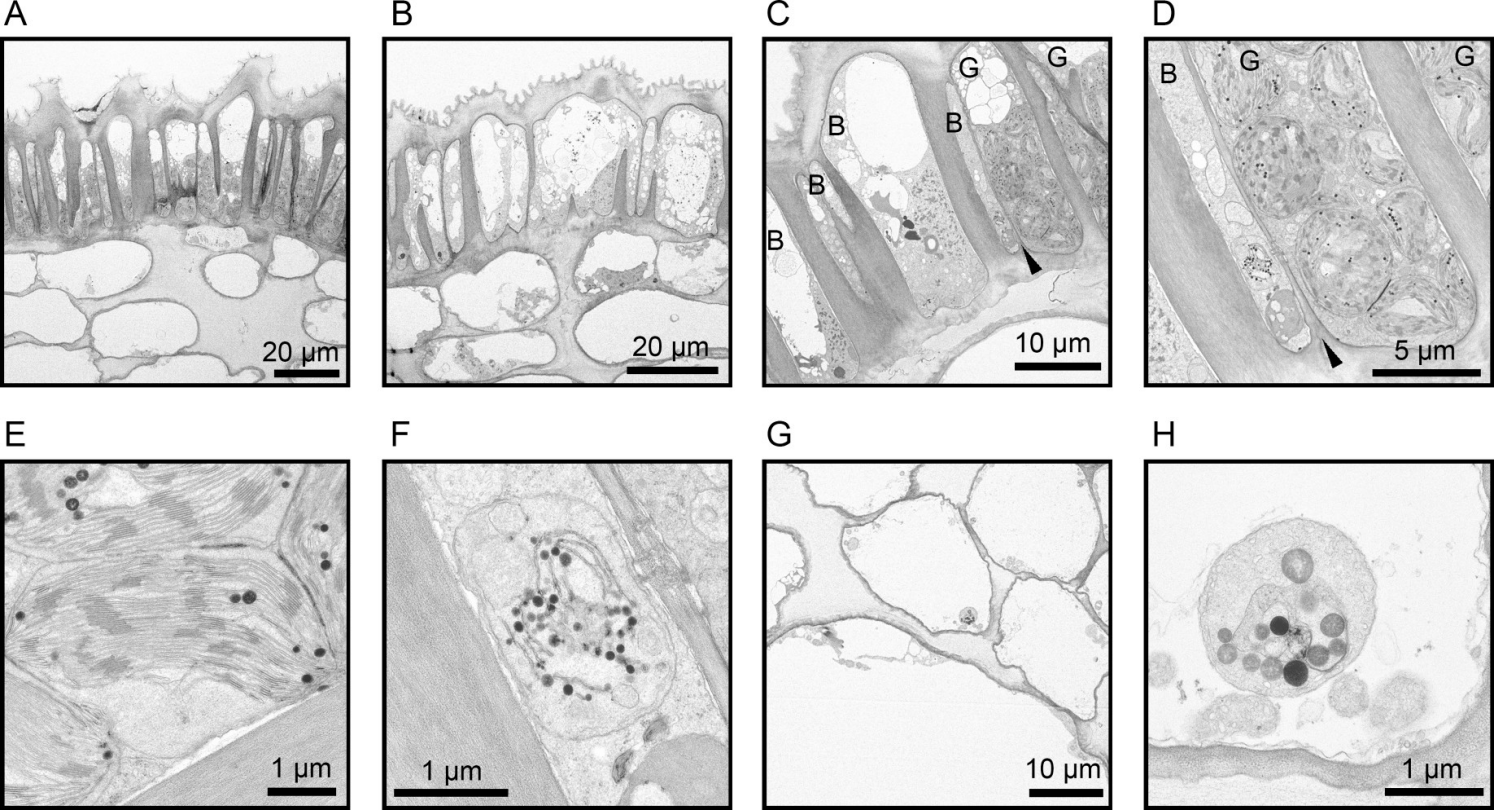
Transmission electron micrographs of the corolla of Japanese gentian ‘Bzc-1’. (A) Cross-section of green epidermal cells (GECs; upper side) and corolla mesophyll cells (lower side), (B) cross-section of blue epidermal cells (BECs) and corolla mesophyll cells, (C, D) cross-section of GECs and BECs. The letters G and B denote GEC and BEC, respectively. Black arrows indicate the boundary between GECs and BECs. (E) Chloroplasts in GECs, (F) plastids in BECs, (G) cross-section of corolla mesophyll cells, (H) plastids in a corolla mesophyll cell.

Given that the green granules in the GECs were approximately 5 μm in diameter and emitted red autofluorescence upon excitation with blue light, we speculated that the structures were chloroplasts. However, it is generally accepted that chloroplasts are absent in epidermal cells. Transmission electron microscopy (TEM) analysis was performed to observe the detailed structure of the green granules in GECs. Fig 2 shows TEM images of cross-sections of the ‘Bzc-1’ corolla. The GECs were elongated in shape, with vacuoles in the upper part of the cell lumen and numerous chloroplasts observed in the lower part of the cell lumen (Fig 2A). The BECs also were elongated in shape similar to GECs, but the cell lumen was mostly occupied by vacuoles and contained plastids instead of chloroplasts (Fig 2B). In addition, observation of the boundary region between GECs and BECs revealed that both cell types were clearly distinguished and no cells of intermediate morphology were observed (Fig 2C and 2D). Fig 2E shows the chloroplast structure of GECs. The chloroplasts contained thylakoids and developed grana, similar to the chloroplasts typically present in the leaves of higher plants. In contrast, in the plastid of BECs, the thylakoids were collapsed and no grana were observed (Fig 2F). In addition, the number of plastoglobules (PGs) was higher in plastids of BECs than those of GECs (Fig 2E and 2F). Elevation in PG number and size is observed during chloroplast degradation under leaf senescence [26]. Therefore, the plastids of BECs are not considered to be chromoplasts associated with flower color but plastid forms after chloroplast degradation. Corolla mesophyll cells showed an elliptic shape, and the cell lumen was almost entirely occupied by vacuoles (Fig 2G). In addition, the plastids of corolla mesophyll cells contained larger PGs (Fig 2H).

### Photosynthesis of the green spots

**Fig 3:**
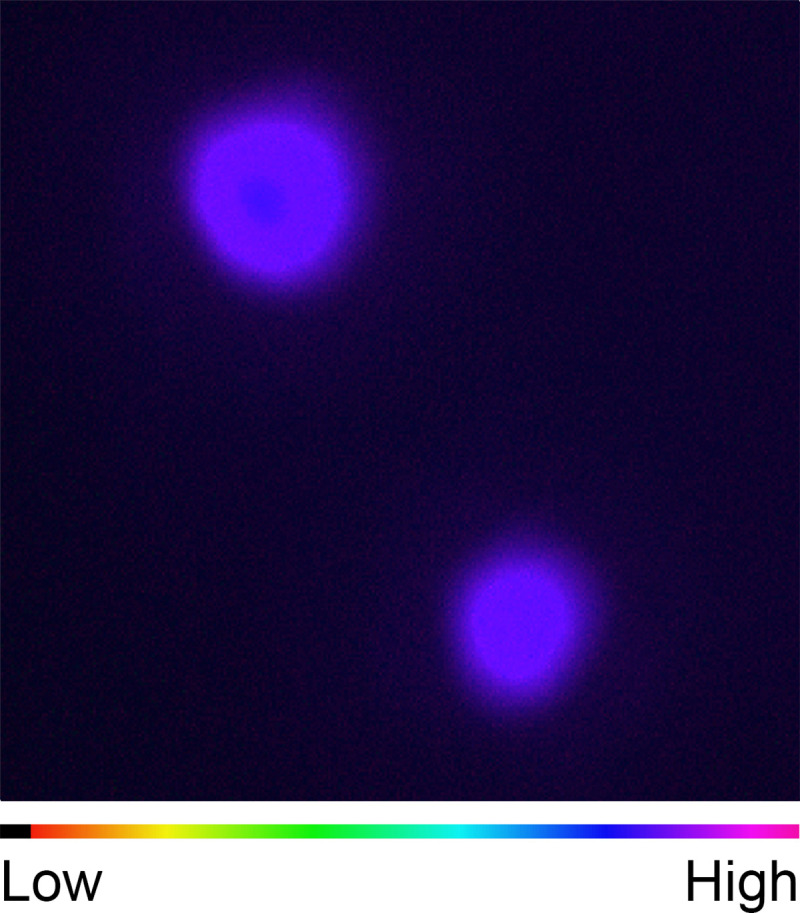
Pseudo-color image of photosynthetic yield of the Japanese gentian ‘Bzc-1’ corolla. Φ_II_ of green spots and neighboring cells was determined under actinic light of 449 μmol photons m^−2^ s^−1^ at room temperature and is shown as a pseudo-color image.

Next, we examined the function of the chloroplasts in GECs. Given that the size of the green spot was quite small, photosynthetic yield of chloroplasts in GECs was determined using the MICROSCOPY version of an Imaging-PAM fluorometer. One to two green spots were obtained from three independent corollas and measured at a total of five points. Under actinic light of 449 μmol m^−2^ s^−1^, which is comparable to the photon flux density in the greenhouse for growing ‘Bzc-1’, areas corresponding to green spots showed relatively uniform and high Φ_II_, a parameter representing the relative yield of photosynthetic electron transport (Fig 3). Photosynthetic activity was not detected in the parts outside of the green spots. The mean Φ_II_ was 0.50 ± 0.01 for the five green spots. This value is comparable to those of *Arabidopsis thaliana* leaves [27] or *Hibiscus rosa*-*sinensis* [28], indicating that the functioning of photosystem II (PSII) and photosystem I (PSI) is intact in the chloroplasts in GECs.

**Fig 4:**
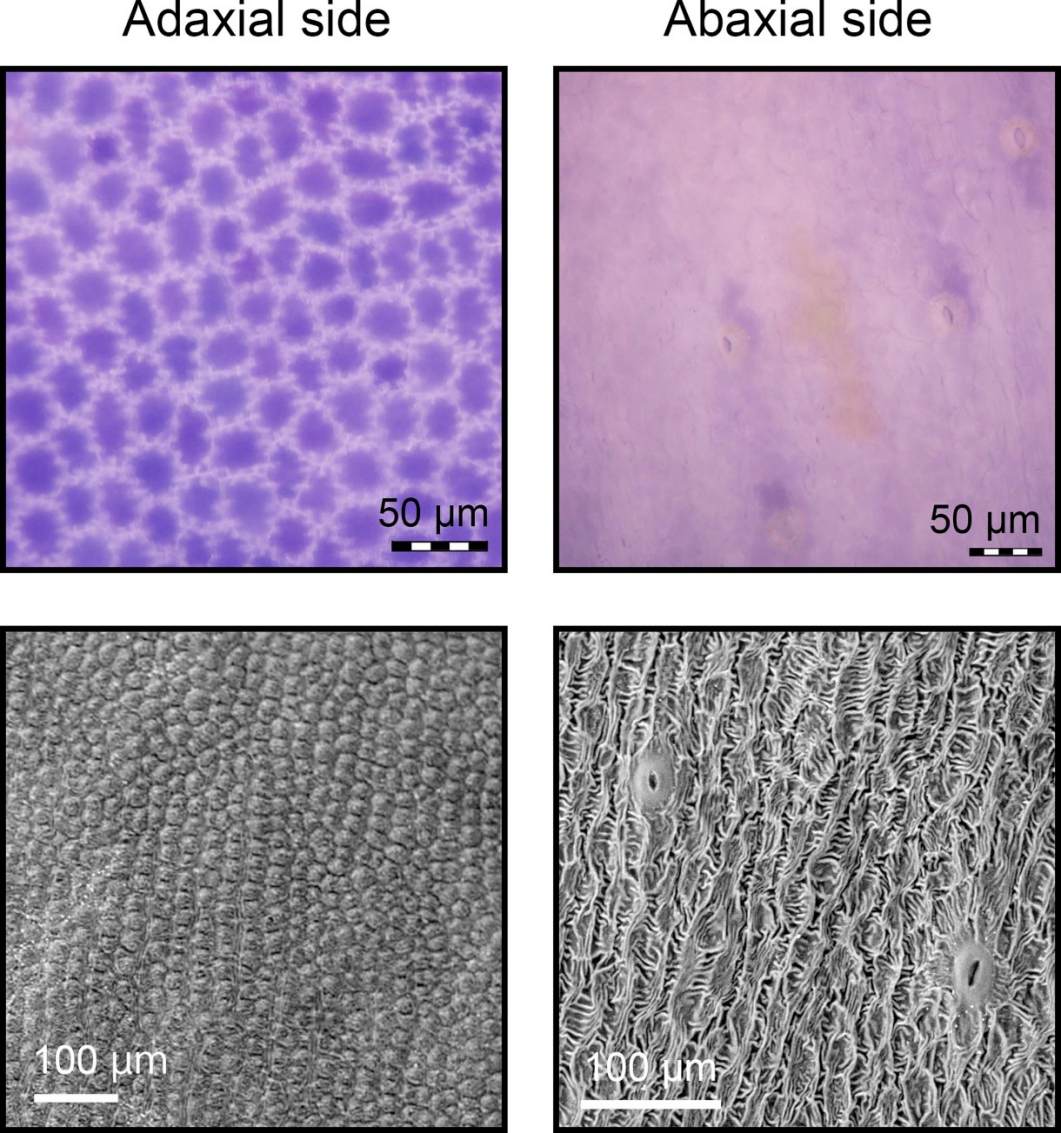
Morphology and distribution of stomata in the corolla of Japanese gentian ‘Bzc-1’. Images of the adaxial and abaxial surfaces of the corolla were acquired using an opto-digital microscope (upper panels) and scanning electron microscope (lower panels).

If these chloroplasts are active in photosynthesis, carbon dioxide should be taken up from the atmosphere into chloroplasts. We observed that stomata were present on the abaxial surface of the corolla but not on the adaxial surface (Fig 4). These results supported the conclusion that the green spots of the ‘Bzc-1’ corolla are photosynthetically active.

### Chloroplast development and degradation in corolla

**Fig 5:**
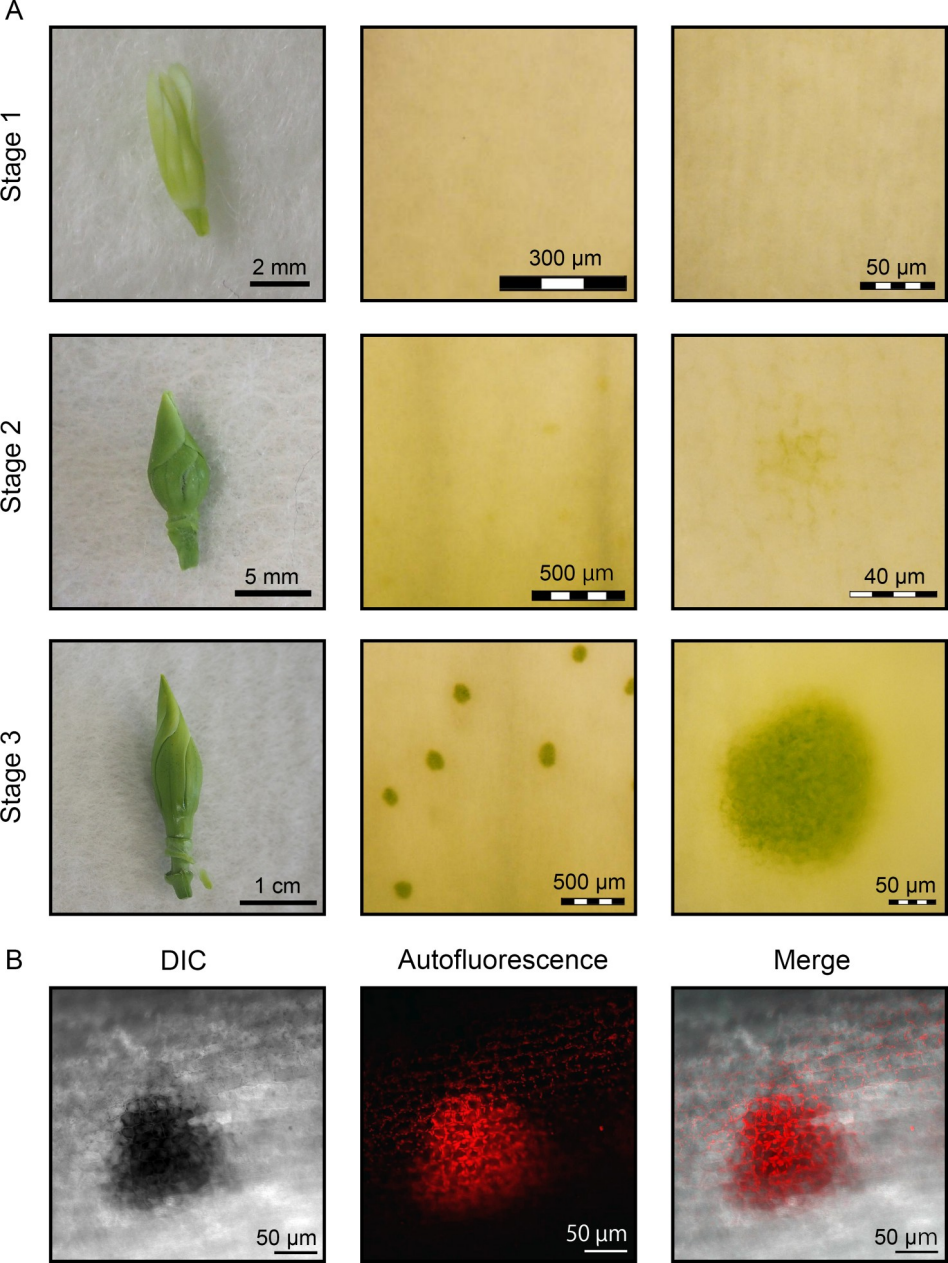
Developmental process of green spots in floral buds of Japanese gentian ‘Bzc-1’. (A) Developmental status of green spots in floral buds at three developmental stages. (B) Chlorophyll autofluorescence from the green spot and epidermal cells of buds (stage 3).

The results thus far revealed that the green spots on the ‘Bzc-1’ corolla were composed of GECs containing functional chloroplasts. It is widely accepted that chloroplasts are absent in epidermal cells except for guard cells in plant leaves. No chloroplasts were observed in epidermal cells of ‘Bzc-1’ leaves (S2 Fig), implying that green spots are formed in the epidermis of the corolla in developing buds after the plant switches from vegetative to reproductive growth. To determine when green spots develop in the ‘Bzc-1’ corolla, the floral bud epidermis at three developmental stages (stages 1, 2, and 3) was observed (Fig 5A). Stage 3 buds possessed well-developed green spots, whereas stage 1 buds showed no green spots. Stage 2 buds had developing green spots consisting of approximately 10 GECs. This observation revealed that green spots developed during bud growth and arose independently of each other.

In stage 3 buds, we detected chlorophyll autofluorescence not only in green spots but also in surrounding epidermal cells (Fig 5B). The size of granules emitting chlorophyll autofluorescence observed in the surrounding epidermal cells was distinctly smaller than GEC chloroplasts. In *Arabidopsis thaliana*, chloroplasts in the corolla are converted to leucoplasts during corolla development [29]. Therefore, during bud development, it is suggested that chloroplast degradation is promoted in epidermal cells except GECs. No chlorophyll fluorescence was observed in BECs (Fig 1J), suggesting that chloroplast degradation was completed in the corolla of fully opened flowers. In addition, we observed chloroplasts in mesophyll cells of stage 3 buds (S2 Fig). Given that chloroplasts are not observed in the corolla mesophyll cells of a fully opened flower (Figs 1E and 2G), it is suggested that these chloroplasts are degraded during development from the bud to anthesis.

## Discussion

In the present study, we revealed that green spots on the ‘Bzc-1’ corolla have functional chloroplasts and perform photosynthesis (Figs 2 and 3). Interestingly, the greens spots were composed of only epidermal cells (Figs 1 and 2). Furthermore, we observed that the ‘Bzc-1’ corolla bears stomata on the abaxial surface (i.e., the opposite surface to that of the green spots) (Fig 4). In leaves, stomata are involved not only in gas exchange with the atmosphere during photosynthesis, but also play a role in lowering the leaf temperature that has risen owing to strong sunlight during transpiration. Previous studies have been conducted on stomata in corollas, focusing on transpiration [30, 31], but to our knowledge no information on photosynthesis is available. The presence of stomata in the ‘Bzc-1’ corolla is suggested to aid in the continued photosynthesis of the green spots. The green spots develop during floral bud growth (Fig 5). The corolla contains two types of epidermal cells: GECs that develop and maintain chloroplasts, and BECs in which chloroplasts are degraded. These findings are apparently unique and Japanese gentian may prove to be ideal material for studying plastid dedifferentiation.

It is an unusual phenomenon that functional chloroplasts are formed and maintained in epidermal cells. In contrast to gentian, green-flowered chrysanthemum (*Chrysanthemum morifolium*) accumulated chloroplasts in mesophyll cells but not in epidermal cells (S3 Fig). It is proposed that green-flowered mutants, such as the green-flowered chrysanthemum, if they arose in nature, may soon disappear because the green flower trait is undesirable for reproduction [32]. However, green spots are considered to perform a function because they are often present in wild-type gentian flowers of various *Gentiana* species [3, 33] and are independent of the corolla color (S1 Fig). Previous studies have noted the existence of chloroplasts in the epidermal pavement cells of *Arabidopsis thaliana* [34–37]. However, the chloroplasts described in these studies are smaller than mesophyll chloroplasts, indicating that these chloroplasts are not equivalent to GEC chloroplasts.

Generally, the energy that supports plant life is provided by photosynthesis in the leaves [38]. On the other hand, it has been reported that photosynthesis performed in tissues other than leaves, in green non-leaf organs (e.g., wheat ear, sycamore seed pods, tomato fruit, strawberry fruit, greengage, cherries and apples), contributes to yield [39]. One possibility is that green spots on the corolla of Japanese gentian contribute to extension of the flower life. However, because the area of green spots on the corolla is small, their importance as a site for energy production must be considered with caution.

An additional possibility is to consider the biological function of the green spots as a floral pattern. It is widely accepted that flower patterns, including spots, act as a nectar guide to attract pollinators [40–42]. In Asiatic hybrid lilies and *Clarkia gracilis*, anthocyanin accumulation is regulated to form splatter-type spots and sector-type spots, respectively [21, 22]. However, it must be taken into account that the green spots of Japanese gentian are dependent on chlorophyll and show different optical properties from spots composed of anthocyanins. *Swertia bimaculata*, a member of the Gentianaceae, uses nectaries of a flat, yellow-green, spot-like form located in the middle of the corolla lobe to attract pollinators [23]. However, compared with the green spots of Japanese gentian, the nectaries of *S*. *bimaculata* are relatively large, few in number (two per corolla lobe), and their location is limited. Therefore, it is unlikely that green spots of Japanese gentians serve the same function. A field investigation is required to evaluate the function of the green spots of Japanese gentians in pollination, e.g., as a possible guide to attract pollinators.

The color of the green spots is derived from chlorophyll. Chlorophyll has a photosensitizing effect, thus it requires a functional chloroplast for safe accumulation. In Japanese gentian, the GECs possessed functional chloroplasts (Figs 2A, 2E and 3). In leaves, two transcription factor families, namely the GATA NITRATE-INDUCIBLE CARBON-METABOLISM-INVOLVED (GNC) family and the GOLDEN TWO-LIKE (GLK) family, play a central role in chloroplast development [43]. In chrysanthemum, expression levels of genes encoding critical enzymes (glutamyl-tRNA reductase, Mg-protoporphyrin IX chelatase, Mg-protoporphyrin IX monomethylester cyclase, and protochlorophyllide oxidoreductase) for chlorophyll synthesis are strongly correlated with chlorophyll content and flower color [44]. Analysis of the expression profiles of these genes in gentian is required in a future study. Elucidation of the regulatory mechanism of plastid differentiation in GECs and BECs is predicted to reveal a novel regulatory system for plastid differentiation. The master regulator of chloroplast development in corolla will be useful for development of molecular markers for green spots in Japanese gentian in the future.

## Supporting information

S1 Fig Flower variation of Japanese gentian cultivated at the Iwate Agricultural Research Center. (TIF)

S2 Fig Morphology of Japanese gentian ‘Bzc-1’. (A) Plant morphology of ‘Bzc-1’ grown in a pot in the greenhouse. (B) Cross-sections of a leaf and a floral bud.
(TIF)

S3 Fig Morphology (left panel) and cross-section (right panel) of the corolla of green-flowered chrysanthemum (*Chrysanthemum morifolium*). Cut flowers of green-flowered chrysanthemum were purchased from a local market.
(TIF)

## Abbreviations

BEC: blue epidermal cell
GEC: green epidermal cell
PAM: pulse-amplitude-modulated
PAR: photosynthetically active radiation
PG: plastoglobule
PSI: photosystem I
PSII: photosystem II
SEM: scanning electron microscope
TEM: transmission electron microscopy
Φ_II_: quantum yield of photosystem II

## References

[1] Davies K, Albert NW, Schwinn KE. From landing lights to mimicry: The molecular regulation of flower colouration and mechanisms for pigmentation patterning. Funct Plant Biol. 2012; 39: 619–638. 10.1071/FP12195 32480814

[2] Sheehan H, Hermann K, Kuhlemeier C. Color and scent: how single genes influence pollinator attraction. Cold Spring Harb Symp Quant Biol. 2012; 77: 117–133. 10.1101/sqb.2013.77.014712 23467550

[3] Köhlein F. Gentians. Timber Press Portland, Oregon, U.S.A.; 1991.

[4] Yang B, Kim S, Kim JH, Lim C, Kim H, Cho S. *Gentiana scabra* Bunge roots alleviates skin lesions of contact dermatitis in mice. J Ethnopharmacol. 2019; 233: 141–147. 10.1016/j.jep.2018.12.046 30630090

[5] Nishihara M, Tasaki K, Sasaki N, Takahashi H. Development of basic technologies for improvement of breeding and cultivation of Japanese gentian. Breed Sci. 2018; 68: 14–24. 10.1270/jsbbs.17074 29681744 PMC5903972

[6] Nakatsuka T, Saito M, Yamada E, Fujita K, Yamagishi N, Yoshikawa N, et al Isolation and characterization of the C-class *MADS-box* gene involved in the formation of double flowers in Japanese gentian. BMC Plant Biol. 2015; 15: 182 10.1186/s12870-015-0569-3 26183329 PMC4504037

[7] Nakatsuka T, Saito M, Nishihara M. Functional characterization of duplicated B-class MADS-box genes in Japanese gentian. Plant Cell Rep. 2016; 35: 895–904. 10.1007/s00299-015-1930-6 26769577

[8] Imamura T, Nakatsuka T, Higuchi A, Nishihara M, Takahashi H. The gentian orthologs of the *FT*/*TFL1* gene family control floral initiation in *Gentiana*. Plant Cell Physiol. 2011; 52: 1031–1041. 10.1093/pcp/pcr055 21531759

[9] Takahashi H, Imamura T, Konno N, Takeda T, Fujita K, Konishi T, et al The gentio-oligosaccharide gentiobiose functions in the modulation of bud dormancy in the herbaceous perennial *Gentiana*. Plant Cell. 2014; 26: 3949–3963. 10.1105/tpc.114.131631 25326293 PMC4247589

[10] Lee JH, Sugawara E, Yokoi S, Takahara Y. Genotypic variation of volatile compounds from flowers gentians. Breed Sci. 2010; 60: 9–17.

[11] Goto T, Kondo T, Tamura H, Imagawa H, Iino A, Takeda K. Structure of gentiodelphin, an acylated anthocyanin from *Gentiana maikinoi*, that is stable in dilute aqueous solution. Tetrahedron Lett. 1982; 23: 3695–3698.

[12] Hosokawa K, Fukushi E, Kawabata J, Fujii C, Ito T, Yamamura S. Seven acylated anthocyanins in blue flowers of *Gentiana*. Phytochemistry. 1997; 45: 167–171. 10.1016/0031-9422(94)00778-r 7766400

[13] Nakatsuka T, Nishihara M, Mishiba K, Hirano H, Yamamura S. Two different transposable elements inserted in flavonoid 3',5'-hydroxylase gene contribute to pink flower coloration in *Gentiana scabra*. Mol Genet Genomics. 2006; 275: 231–241. 10.1007/s00438-005-0083-7 16362368

[14] Nishihara M, Hikage T, Yamada E, Nakatsuka T. A single-base substitution suppresses flower color mutation caused by a novel miniature inverted-repeat transposable element in gentian. Mol Genet Genomics. 2011; 286: 371–382. 10.1007/s00438-011-0652-x 22002873

[15] Nakatsuka T, Nishihara M, Mishiba K, Yamamura S. Two different mutations are involved in the formation of white-flowered gentian plants. Plant Sci. 2005; 169: 949–958. 10.1016/j.plantsci.2005.06.013

[16] Nakatsuka T, Haruta KS, Pitaksutheepong C, Abe Y, Kakizaki Y, Yamamoto K, et al Identification and characterization of R2R3-MYB and bHLH transcription factors regulating anthocyanin biosynthesis in gentian flowers. Plant Cell Physiol. 2008; 49: 1818–1829. 10.1093/pcp/pcn163 18974195

[17] Nakatsuka T, Saito M, Sato-Ushiku Y, Yamada E, Nakasato T, Hoshi N, et al Development of DNA markers that discriminate between white- and blue-flowers in Japanese gentian plants. Euphytica. 2011; 184: 335–344. 10.1007/s10681-011-0534-7

[18] Kakizaki Y, Nakatsuka T, Kawamura H, Abe J, Abe Y, Yamamura S, et al Development of codominant DNA marker distinguishing pink from blue flowers in *Gentiana scabra*. Breed Res. 2009; 11: 9–14. 10.1270/jsbbr.11.9

[19] Sasaki N, Watanabe A, Asakawa T, Sasaki M, Hoshi N, Naito Z, et al Evaluation of the biological effect of ion beam irradiation on perennial gentian and apple plants. Plant Biotechnol. 2018; 35: 249–257. 10.5511/plantbiotechnology.18.0612a PMC6879364 31819730

[20] Tasaki K, Higuchi A, Watanabe A, Sasaki N, Nishihara M. Effects of knocking out three anthocyanin modification genes on the blue pigmentation of gentian flowers. Sci Rep. 2019; 9: 15831 10.1038/s41598-019-51808-3 31676875 PMC6825144

[21] Yamagishi M, Toda S, Tasaki K. The novel allele of the *LhMYB12* gene is involved in splatter-type spot formation on the flower tepals of Asiatic hybrid lilies (*Lilium* spp.). New Phytol. 2014; 201: 1009–1020. 10.1111/nph.12572 24180488

[22] Martins TR, Berg JJ, Blinka S, Rausher MD, Baum DA. Precise spatio-temporal regulation of the anthocyanin biosynthetic pathway leads to petal spot formation in *Clarkia gracilis* (Onagraceae). New Phytol. 2013; 197: 958–969. 10.1111/nph.12062 23231386 PMC3540125

[23] Wang S, Fu WL, Du W, Zhang Q, Li Y, Lyu YS, et al Nectary tracks as pollinator manipulators: The pollination ecology of *Swertia bimaculata* (Gentianaceae). Ecol Evol. 2018; 8: 3187–3207. 10.1002/ece3.3838 29607017 PMC5869268

[24] Tasaki K, Higuchi A, Fujita K, Watanabe A, Sasaki N, Fujiwara K, et al Development of molecular markers for breeding of double flowers in Japanese gentian. Mol Breed. 2017; 37 10.1007/s11032-017-0633-9

[25] Bailes EJ, Glover BJ. Intraspecific variation in the petal epidermal cell morphology of *Vicia faba* L. (Fabaceae). Flora. 2018; 244–245: 29–36. 10.1016/j.flora.2018.06.005 30008511 PMC6039855

[26] Tamary E, Nevo R, Naveh L, Levin-Zaidman S, Kiss V, Savidor A, et al Chlorophyll catabolism precedes changes in chloroplast structure and proteome during leaf senescence. Plant Direct. 2019; 3: e00127 10.1002/pld3.127 31245770 PMC6508775

[27] Wiciarz M, Gubernator B, Kruk J, Niewiadomska E. Enhanced chloroplastic generation of H_2_O_2_ in stress-resistant *Thellungiella salsuginea* in comparison to *Arabidopsis thaliana*. Physiol Plant. 2015; 153: 467–476. 10.1111/ppl.12248 24961163 PMC4359041

[28] Paredes M, Quiles MJ. The Effects of Cold Stress on Photosynthesis in Hibiscus Plants. PLoS One. 2015; 10: e0137472 10.1371/journal.pone.0137472 26360248 PMC4567064

[29] Pyke KA, Page AM. Plastid ontogeny during petal development in *Arabidopsis*. Plant Physiol. 1998; 116: 797–803. 10.1104/pp.116.2.797 9489024 PMC35139

[30] Roddy AB, Brodersen CR, Dawson TE. Hydraulic conductance and the maintenance of water balance in flowers. Plant Cell Environ. 2016; 39: 2123–2132. 10.1111/pce.12761 27144996

[31] Zhang FP, Carins Murphy MR, Cardoso AA, Jordan GJ, Brodribb TJ. Similar geometric rules govern the distribution of veins and stomata in petals, sepals and leaves. New Phytol. 2018; 219: 1224–1234. 10.1111/nph.15210 29761509

[32] Ohmiya A. Molecular mechanisms underlying the diverse array of petal colors in chrysanthemum flowers. Breed Sci. 2018; 68: 119–127. 10.1270/jsbbs.17075 29681754 PMC5903973

[33] Ho TN, Liu SW. A worldwide monograph of *Gentiana*.: Science Press, Beijing, China; 2001.

[34] Barton KA, Schattat MH, Jakob T, Hause G, Wilhelm C, McKenna JF, et al Epidermal Pavement Cells of Arabidopsis Have Chloroplasts. Plant Physiol. 2016; 171: 723–726. 10.1104/pp.16.00608 27288524 PMC4902630

[35] Fujiwara MT, Kojo KH, Kazama Y, Sasaki S, Abe T, Itoh RD. The *Arabidopsis minE* mutation causes new plastid and FtsZ1 localization phenotypes in the leaf epidermis. Front Plant Sci. 2015; 6: 823 10.3389/fpls.2015.00823 26500667 PMC4593956

[36] Pyke KA, Leech RM. A Genetic Analysis of Chloroplast Division and Expansion in *Arabidopsis thaliana*. Plant Physiol. 1994; 104: 201–207. 10.1104/pp.104.1.201 12232072 PMC159178

[37] Robertson EJ, Rutherford SM, Leech RM. Characterization of chloroplast division using the *Arabidopsis* mutant arc5. Plant Physiol. 1996; 112: 149–159. 10.1104/pp.112.1.149 8819323 PMC157934

[38] Niyogi KK. Safety valves for photosynthesis. Curr Opin Plant Biol. 2000; 3: 455–460. 10.1016/s1369-5266(00)00113-8 11074375

[39] Simkin AJ, Faralli M, Ramamoorthy S, Lawson T. Photosynthesis in non-foliar tissues: implications for yield. Plant J. 2020; 101: 1001–1015. 10.1111/tpj.14633 31802560 PMC7064926

[40] Johnson S, Midgley J. Fly pollination of *Gorteria diffusa* (Asteraceae), and a possible mimetic function for dark spots on the capitulum. Am J Bot. 1997; 84: 429 21708596

[41] Van Kleunen M, Nanni I, Donaldson JS, Manning JC. The role of beetle marks and flower colour on visitation by monkey beetles (hopliini) in the greater cape floral region, South Africa. Ann Bot. 2007; 100: 1483–1489. 10.1093/aob/mcm256 17951585 PMC2759239

[42] Leonard AS, Papaj DR. ‘X’ marks the spot: The possible benefits of nectar guides to bees and plants. Funct Ecol. 2011; 25: 1293–1301.

[43] Zubo YO, Blakley IC, Franco-Zorrilla JM, Yamburenko MV, Solano R, Kieber JJ, et al Coordination of Chloroplast Development through the Action of the GNC and GLK Transcription Factor Families. Plant Physiol. 2018; 178: 130–147. 10.1104/pp.18.00414 30002259 PMC6130010

[44] Ohmiya A, Sasaki K, Nashima K, Oda-Yamamizo C, Hirashima M, Sumitomo K. Transcriptome analysis in petals and leaves of chrysanthemums with different chlorophyll levels. BMC Plant Biol. 2017; 17: 202 10.1186/s12870-017-1156-6 29141585 PMC5688696

