# Supplementary figures and images for: Morphological and cytological observations of corolla green spots reveal the presence of functional chloroplasts in Japanese gentian
Source: PLoS One. 2020 Aug 26;15(8):e0237173. doi: 10.1371/journal.pone.0237173 (PMC7449470; doi:10.1371/journal.pone.0237173)

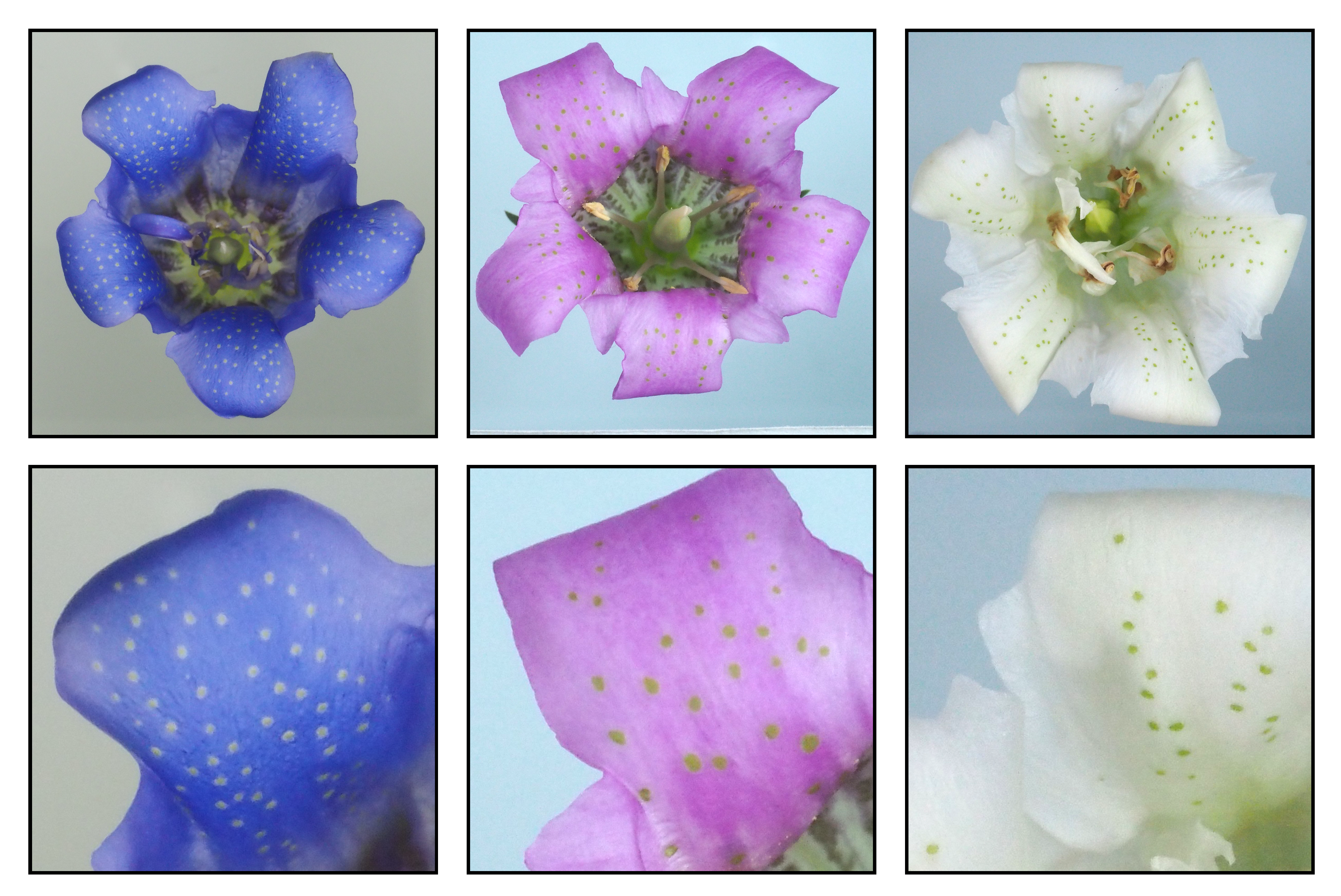

Supplement: S1 Fig — (TIF) [file pone.0237173.s001.tif]

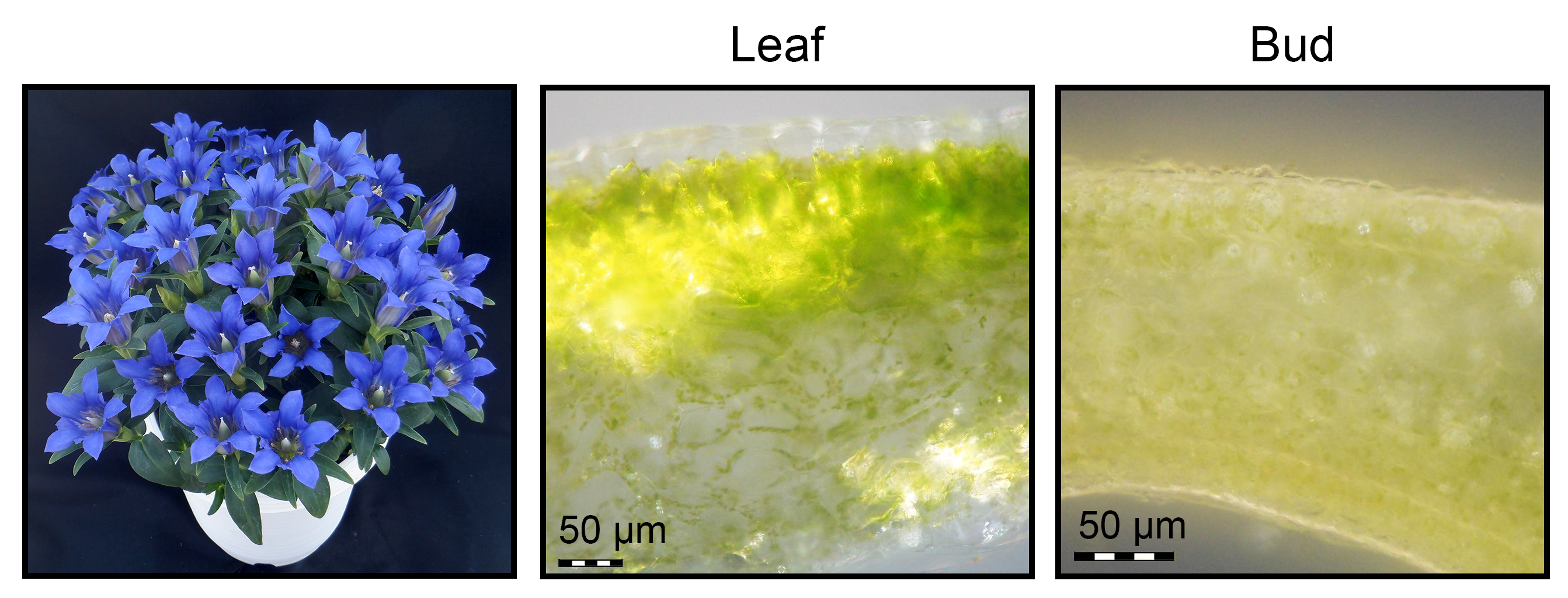

Supplement: S2 Fig — (A) Plant morphology of ‘Bzc-1’ grown in a pot in the greenhouse. (B) Cross-sections of a leaf and a floral bud. (TIF) [file pone.0237173.s002.tif]

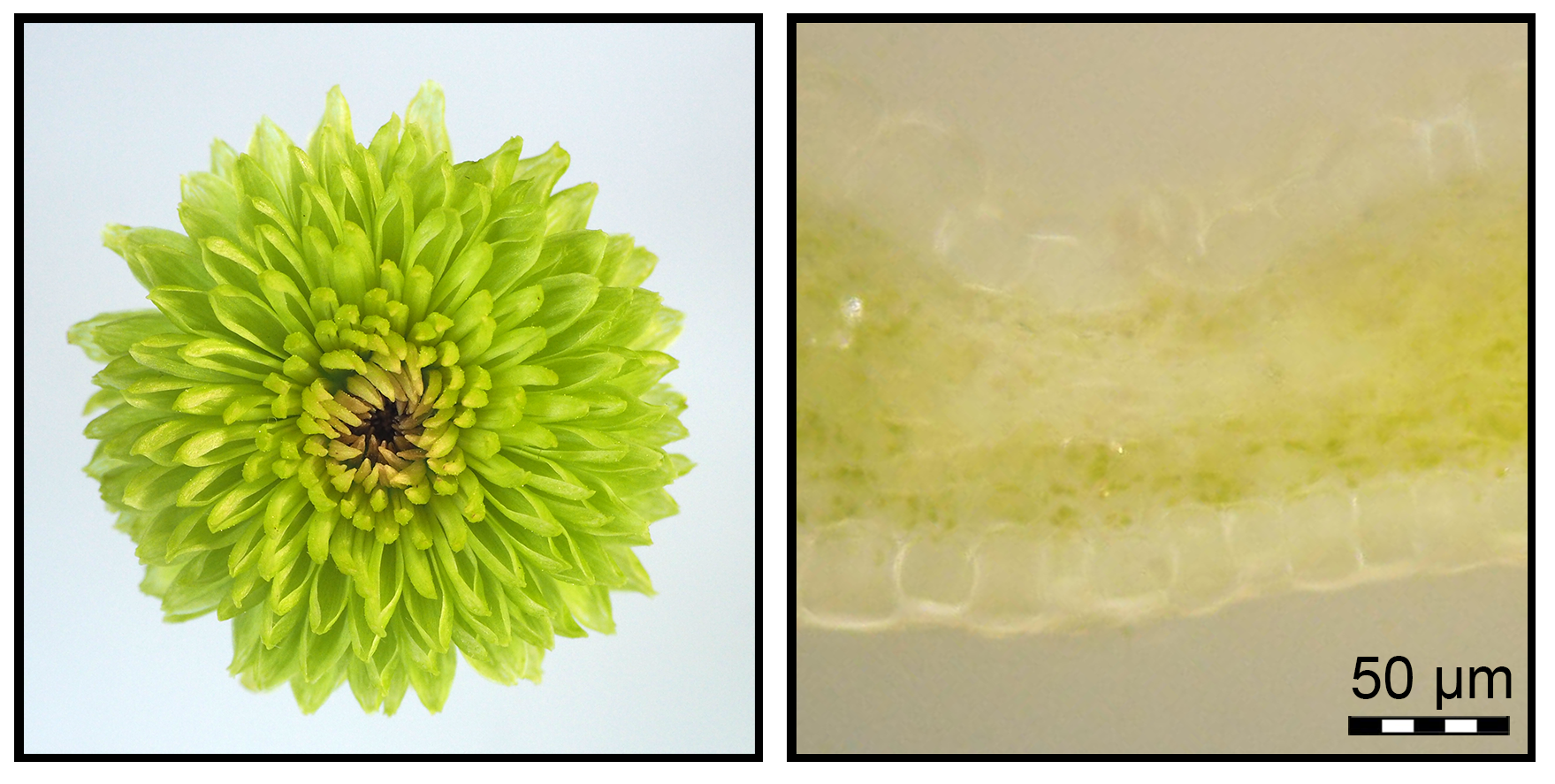

Supplement: S3 Fig — Cut flowers of green-flowered chrysanthemum were purchased from a local market. (TIF) [file pone.0237173.s003.tif]
